# Improved Osprey Optimization Algorithm with Multi-Strategy Fusion

**DOI:** 10.3390/biomimetics9110670

**Published:** 2024-11-01

**Authors:** Wenli Lei, Jinping Han, Xinghao Wu

**Affiliations:** College of Physics and Electronic Information, Yan’an University, Yan’an 716000, China; jpinghan@yau.edu.cn (J.H.); wxh1@yau.edu.cn (X.W.)

**Keywords:** osprey optimization algorithm, Fuch chaotic mapping, adaptive weighting factor, Cauchy’s variation

## Abstract

The osprey optimization algorithm (OOA) is an effective metaheuristic algorithm. Although the OOA has the characteristics of strong optimality-seeking ability and fast convergence speed, it also has the disadvantages of imbalance between global exploration and local exploitation ability, easily falling into local optima in the later stage, and reduced population diversity and convergence speed. Therefore, this paper proposes an improved osprey optimization algorithm (IOOA) with multi-strategy fusion. First, Fuch chaotic mapping is used to initialize the ospreys’ population and increase the population diversity. Then, an adaptive weighting factor is introduced in the exploration phase of the algorithm to help the algorithm improve the convergence accuracy. The Cauchy variation strategy is integrated in the algorithm’s exploitation stage to enhance the diversity of the ospreys’ population and avoid falling into local optima. Finally, a Warner mechanism for the sparrow search algorithm is introduced to coordinate the algorithm’s local optimization and global search capabilities. The IOOA with various optimization algorithms is tested in a simulation for 10 benchmark test functions and 15 CEC2017 test functions, and non-parametric tests are performed on the IOOA. Experimental results show that the IOOA achieves improved accuracy and stability. The application of the IOOA to the three-bar truss engineering design problem further verifies its superiority in dealing with practical optimization problems.

## 1. Introduction

Due to their simplicity, flexibility, and efficiency, swarm intelligence algorithms have been widely used in recent years in engineering fields such as cluster task planning [1,2], workshop scheduling [3,4], power system optimization [5,6], and path planning [7,8]. The osprey optimization algorithm (OOA) [9], first proposed in 2023 by Mohammad Dehghani and Pavel Trojovský, is a novel bio-heuristic optimization algorithm that simulates the hunting–predatory behavior of ospreys in nature. Based on the simulation of ospreys’ hunting process, the OOA establishes a mathematical model of the two phases of ospreys’ exploration and exploitation. The algorithm has the advantages of simple structure, a robust global search capability, and fast convergence. However, it inevitably encounters common problems arising from metaheuristic algorithms, such as reduced convergence speed of algorithm iterations, a tendency to fall into local optimality, etc.

In order to improve the convergence speed of algorithms and enhance the ability of algorithms to leap out of local extremes, many scholars have conducted extensive research in this field. The study in Yu et al. [10] improved the gray wolf optimization algorithm by using the good point-set method to initialize the population to increase the population diversity and introducing a beetle tentacle search mechanism to prevent the algorithm from falling into local optima. The study in [11] proposed an ADFPSO algorithm by using a fitness-based driver to improve the development capability of the PSO algorithm and a novelty-based driver to enhance the exploration capability of the PSO algorithm and by introducing an adaptive weighting factor to coordinate the weights of the two drivers at different stages of the optimization search. The study in [12] proposed a improved gorilla troops optimizer based on lens opposition-based learning and adaptive hill climbing for global optimization, using reverse convex lens imaging learning to expand the search range and avoid falling into local optima and introducing an adaptive hill climbing algorithm in combination with GTO to improve the solution accuracy. The study in [13] proposed a whale optimization algorithm based on the siege mechanism, which combines the siege mechanism of the Harris hawk optimization algorithm to improve the global exploration and local optimization-seeking ability of the whale optimization algorithm. The study in [14] solved the problem of low solution accuracy and the poor stability of the Marine Predator Algorithm (MPA) by introducing an elite reverse learning strategy and a golden sine strategy. The study in [15] proposed an improved grasshopper optimization algorithm (IWGOA) that introduced the weed algorithm and random wandering strategy into the locust algorithm to improve the convergence accuracy. The study in [16] introduced time delay and sorting parameters based on the artificial bee colony algorithm and used chaotic systems to solve the multidimensionalization problem of parameter estimation to prevent the execution process from falling into local optima. The study in [17] proposed a chaotic chimp optimization algorithm based on adaptive tuning, which employs a Sin operator for population initialization to enhance the population richness and also improves the convergence factor (f) and dynamically adjusts the number of chimp precedence echelons, which enhances the algorithm’s global search and local exploitation abilities. The study in [18] improved the osprey optimization algorithm by using Sobol sequences for population initialization, introducing a step factor based on the Weibull distribution to balance the algorithm’s local and global optimality-seeking ability and incorporating firefly perturbations to prevent the algorithm from falling into a local optimum. The study in [19] proposed an attack–defense strategy-assisted osprey optimization algorithm (ADSOOA), which integrates an attack–defense strategy to improve convergence performance and prevent the algorithm from falling into local optima, and applied it to PEMFC parameter identification.

The improvements proposed above for the swarm intelligence algorithm reduce the possibility of the algorithm falling into local extremes to a certain extent, but there are still problems, such as low convergence accuracy and limited improvement in algorithm performance. To better improve the optimization performance and application capability of the osprey optimization algorithm, this paper proposes an improved osprey optimization algorithm (IOOA) with multi-strategy fusion. The algorithm uses Fuch chaotic mapping to make the initialized population more evenly distributed and increase population diversity. The introduction of adaptive weighting factors in the exploration phase of the algorithm improves the convergence speed of the algorithm. Incorporating the Cauchy variation operator during the algorithm’s exploration phase enhances the diversity of the ospreys’ population while improving the algorithm’s ability to leapfrog local extremes. A Warner mechanism is introduced for the sparrow search algorithm to balance the algorithm’s ability to explore globally and develop locally.

## 2. OOA

The OOA is a heuristic algorithm that models the foraging process of osprey populations and consists of the following two parts: an exploration phase and an exploitation phase. First, the osprey population is initialized in the search space with the expression shown in Equation (1).

(1) $$X_{i,j}=lb_{j}+r_{i,j}\cdot (ub_{j}-lb_{j}),i=1,2,\ldots ,N;j=1,2,\ldots ,D$$

where $X_{i,j}$ is the initial position of the *i*th osprey in the *j*th dimension; $lb_{j}$ and $ub_{j}$ are the upper and lower bounds of the *j*th problem variable, respectively; $r_{i,j}$ is a random number in the range of [0, 1]; *N* is the osprey population size; and *D* is the dimension of the problem.

Equation (2) shows the function to calculate the fitness value.

(2) $$F_{i}=F\left(X_{i}\right),i=1,2,\ldots ,N$$

where $F_{i}$ is the fitness value of the *i*th osprey and $X_{i}$ is the position of the *i*th osprey. In this paper, we need to solve for the minimum value of $F_{i}$. The smaller the value, the better it is for the location of the osprey.

After population initialization, the osprey enters the exploration phase, which is also the global exploration phase. Other ospreys’ position with better fitness values in the search space were considered as fish positions. Equation (3) expresses the location of the school of fish for each osprey.

(3) $$FP_{i}=\left\{X_{k}\left|k\in \left\{1,2,\ldots ,N\right\}\cap F_{k}<F_{i}\right\}\cup \left\{X_{best}\right\},i=1,2,\ldots ,N\right.$$

where $FP_{i}$ is the set of fish locations of the *i*th osprey and $X_{best}$ is the location of the osprey with the best fitness value.

In the search space, the osprey randomly selects a fish and attacks it. During the simulation of the movement of the osprey towards the fish, this paper uses Equations (4) and (5) to calculate the new osprey’s position.

(4) $$X_{i,j}^{P1}=X_{i,j}+r_{i,j}\cdot \left(SF_{i,j}-I_{i,j}\cdot X_{i,j}\right),i=1,2,\ldots ,N;j=1,2,\ldots ,D$$

(5) $$X_{i,j}^{P1}=\left\{\begin{array}{c} X_{i,j}^{P1},lb_{j}\leq X_{i,j}^{P1}\leq ub_{j}\\ lb_{j},X_{i,j}^{P1}<lb_{j}\\ ub_{j},X_{i,j}^{P1}>ub_{j} \end{array}\right.$$

where $X_{i}^{P1}$ is the new position of the *i*th osprey in first stage in the *j*th dimension; $SF_{i,j}$ is the fish selected by the *i*th osprey in the *j*th dimension; $r_{i,j}$ is a random number in the range [0, 1]; and $I_{i,j}$ is a random integer, either 1 or 2.

If the new position’s fitness value is better, then the new position replaces the original position; otherwise, it does not. Equation (6) shows the process.

(6) $$X_{i}=\left\{\begin{array}{c} X_{i}^{P1},F_{i}^{P1}<F_{i}\\ X_{i},F_{i}^{P1}\geq F_{i} \end{array}\right.$$

where $X_{i}^{P1}$ is the new position of the *i*th osprey after the first stage update and $F_{i}^{P1}$ is the new position’s fitness value of the *i*th osprey after the first stage update.

After hunting a fish in nature, the osprey will take it to a safe place and feed on it. In this process, this paper uses Equations (7) and (8) to calculate a new random position as the feeding position.

(7) $$X_{i,j}^{P2}=X_{i,j}+\frac{lb_{j}+r_{i,j}\cdot \left(ub_{j}-lb_{j}\right)}{t},i=1,2,\ldots ,N;j=1,2,\ldots ,D;t=1,2,\ldots ,T$$

(8) $$X_{i,j}^{P2}=\left\{\begin{array}{c} X_{i,j}^{P2},lb_{j}\leq X_{i,j}^{P2}\leq ub_{j}\\ lb_{j},X_{i,j}^{P2}<lb_{j}\\ ub_{j},X_{i,j}^{P2}>ub_{j} \end{array}\right.$$

where $X_{i,j}^{P2}$ is the new position of the *i*th osprey in the *j*th dimension in the second stage; $r_{i,j}$ is a random number in the range [0, 1]; *t* is the current number of iterations; and *T* is the maximum number of iterations.

If the new position’s fitness value is better, then the new position replaces the original position; otherwise, it does not. Equation (9) shows the process.

(9) $$X_{i}=\left\{\begin{array}{c} X_{i}^{P2},F_{i}^{P2}<F_{i}\\ X_{i},F_{i}^{P2}\geq F_{i} \end{array}\right.$$

where $X_{i}^{P2}$ is the new position of the *i*th osprey after the second stage update and $F_{i}^{P2}$ is the new position’s fitness value of the *i*th osprey after the second stage update.

## 3. IOOA

### 3.1. Fuch Chaotic Mapping

**Figure 1:**
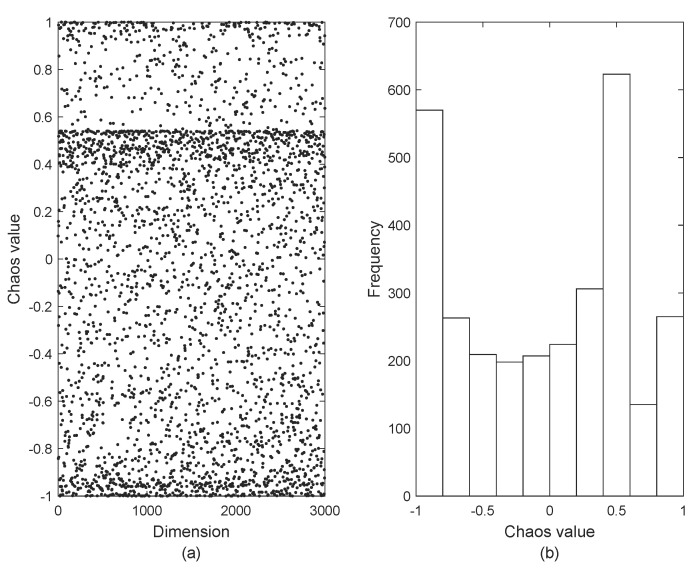
Fuch chaotic distribution.

The OOA initializes the population using random initialization, which makes the population initialization unevenly distributed and leads to a reduction in the initialized population diversity. Chaotic mapping has the advantages of randomness, ergodicity and regularity, which can enrich the population initialization diversity, enhance the global search ability, and improve the algorithm solution’s effectiveness. Therefore, this paper introduces Fuch chaotic mapping [20] to initialize the ospreys’ population, which is a kind of infinitely collapsible chaotic mapping, showing advantages such as stronger chaotic properties and more balanced traversal than the traditional chaotic mapping, and its chaotic sequence is shown in Figure 1. Equation (10) shows the mathematical expression of Fuch chaotic mapping.

(10) $$y_{i+1}=\cos\left(\frac{1}{y_{i}^{2}}\right),y_{i}\in \left(-1,1\right),y_{i}\neq 0,i\in Z^{+}$$

This paper use Equation (10) to generate chaotic variables, and the initialization formula after adding chaotic variables is shown in Equation (11).

(11) $$X_{i,j}=lb_{j}+y_{i}\cdot \left(ub_{j}-lb_{j}\right),i=1,2,\ldots ,N;j=1,2,\ldots ,D$$

where Figure 1a shows the distribution of Fuch chaotic sequences and Figure 1b shows the histogram of the distribution of chaotic sequences.

### 3.2. Adaptive Weighting Factor

The global search ability and local optimization ability of coordinated metaheuristic algorithms are important factors that affect the algorithms’ optimization accuracy and speed. In the first stage of osprey position update, this paper introduces an adaptive weight factor to dynamically regulate the algorithm’s local exploitation and global exploration capabilities, and to improve the algorithm’s optimization accuracy and convergence speed. Equation (12) shows the adaptive weighting factor.

(12) $$w=\frac{e^{\frac{t}{T}}-1}{e-1}$$

At the beginning of the algorithm iterations, the value is small, and the osprey individual focuses more on exploring other locations at this time, which facilitates better global exploration. The w-value increases adaptively in the later iterations, and the osprey individual gradually switches from exploring other locations to exploiting its own neighborhood locations, improving the algorithm’s local exploitation capability. Equation (13) shows the improved osprey position update formula.

(13) $$X_{i,j}^{P1}=w\cdot X_{i,j}+r_{i,j}\cdot \left(SF_{i,j}-I_{i,j}\cdot X_{i,j}\right),i=1,2,\ldots ,N;j=1,2,\ldots ,D$$

### 3.3. Cauchy Variation Strategy

In order to improve the individuals’ quality, increase population diversity, and prevent the algorithms from falling into local optima, this paper incorporates the Cauchy variation strategy in the second stage of the osprey population position update. The one-dimensional standard Cauchy distribution probability density function expression is shown in Equation (14).

(14) $$f\left(x\right)=\frac{1}{\pi }\left(\frac{1}{x^{2}+1}\right),\;-\infty <x<\infty$$

**Figure 2:**
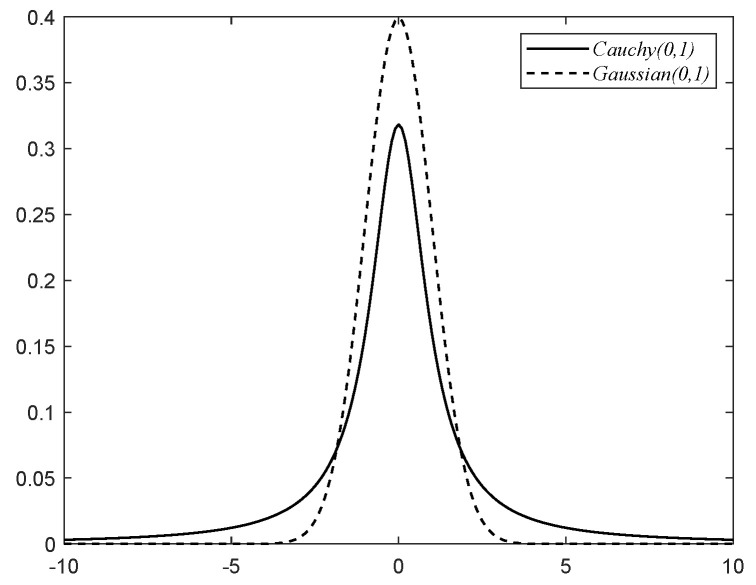
One-dimensional standard Cauchy and Gaussian distribution probability density curves.

Figure 2 shows the probability density curves of the Gaussian and Cauchy distributions. From Figure 2, the Cauchy distribution peak value is smaller than the one-dimensional Gaussian distribution peak value at the origin. The Cauchy distribution ends are flatter and slower than the Gaussian distribution as they approach zero. Thus, the Cauchy distribution can generate larger perturbations. In this paper, the algorithm’s position update formula introduces the Cauchy variation, which enhances the ospreys’ population diversity and improves the algorithm’s global optimization capability.

In the second phase of the IOOA’s population position update, each iteration compares the magnitude of the current osprey’s fitness value in relation to the population average fitness value. When the osprey’s fitness value is lower than the population mean fitness value, it indicates that the current osprey is aggregated. At this point, the perturbation power of the Cauchy operator is exploited to increase the ospreys’ population diversity. When the osprey’s fitness value is higher than the population average fitness value, the OOA position update method is used. Equation (15) shows the improved position update formula.

(15) $$X_{i,j}^{P2}=\left\{\begin{array}{c} X_{best}+X_{best}\cdot \mathrm{cauchy}\left(0,1\right),F_{i}<F_{a\nu g}\\ X_{i,j}+\frac{lb_{j}+r_{i,j}\cdot \left(ub_{j}-lb_{j}\right)}{t},F_{i}\geq F_{a\nu g} \end{array}\right.$$

where cauchy(0,1) denotes denotes the generation of numbers that follow the standard Cauchy distribution, and $F_{a\nu g}$ denotes the population average fitness value.

### 3.4. Integration of the Sparrow Search Algorithm Warner Mechanism

In order to better balance the OOA’s global exploration and local exploitation capabilities, the second stage position update for the ospreys’ population uses the Sparrow Search Algorithm [21] Warner mechanism. Equation (16) shows the improved osprey position update formula.

(16) $$\begin{array}{c} X_{i,j}^{P2}=\left\{\begin{array}{c} X_{best}+\beta \cdot \left|X_{i,j}-X_{best}\right|,F_{i}>F_{g}\\ X_{i,j}+K\cdot \left|\frac{\left|X_{i,j}-X_{worst}\right|}{\left(F_{i}-F_{w}\right)+\varepsilon }\right|,F_{i}=F_{g} \end{array}\right. \end{array}$$

where $F_{i}$ is the current osprey’s fitness value; $F_{g}$ and $F_{w}$ are the current global optimal and worst fitness values; $X_{worst}$ is the current global worst position; β is a random number that satisfies a normal distribution, *K* is a uniform random number in the range [−1, 1]; and ε is the smallest constant. When $F_{i}>F_{g}$, it indicates that the osprey is located at the population edge; this position is not suitable for the osprey to feed and it is easily be attacked by its natural enemies. When $F_{i}=F_{g}$, it indicates that the osprey of the population center senses danger and needs to move closer to other ospreys to reduce the risk of being attacked.

### 3.5. Overall Flow of the IOOA

Step 1: Set the population size *N*; maximum iterations *T*; problem dimension *D*; and boundary condition $lb_{j}$ and $ub_{j}$.

Step 2: Use Fuch mapping to initialize the osprey population and calculate the osprey population fitness values.

Step 3: Calculate the first stage position according to Equation (12).

Step 4: Update $X_{i}$ according to Equation (6).

Step 5: Calculate the second stage position according to Equation (14).

Step 6: Update $X_{i}$ according to Equation (9) and calculate the worst fitness value and its corresponding position.

Step 7: Calculate the second stage position according to Equation (15).

Step 8: Update $X_{i}$ according to Equation (9).

Step 9: Judge whether it reaches the maximum iterations; if so, proceed to the next step, and otherwise, skip to step 2.

Step 10: The procedure ends with the output of the optimal solution.

### 3.6. Time Complexity Analysis

The time complexity is an important metric for evaluating its solution speed. This paper performs time complexity analysis of the IOOA. This paper sets the ospreys’ population size to *N*, the maximum iterations to *T*, and the problem dimension to *D*. In the OOA, each iteration of population initialization takes *O(N × D)*, and both phases of the position update process take *O(N × D × T)*, with the total time complexity of *O(N × D × (1 + 2T))* For the IOOA, the time complexity of population initialization is *O(N×D)*, and both phases of the position update process for introducing the update strategy take *O(N × D × T)*, giving the total time complexity of *O(N × D × (1+2T))*. Thus, the IOOA is equal to the OOA in time complexity; it does not increase the complexity overall and it does not increase the computational burden.

## 4. Simulation Experiments and Result Analysis

### 4.1. Experimental Environment and Test Functions

The simulation environment of this paper is Windows 11 (64-bit) operating system, with an Intel(R) Core(TM) i5-12500H CPU (Intel, Santa Clara, CA, USA) with 3.10 GHz main frequency and 16 GB RAM, and the program is implemented with MatlabR2022a programming. This paper selects 10 benchmark test functions and 15 functions of the CEC2017 test set for optimization-seeking tests. Table 1 shows the benchmark test functions, where F1–F7 are single-peak functions and F8–F10 are multi-peak functions, and Table 2 shows the CEC2017 test functions. To verify the superior performance of the IOOA, Golden Jackal Optimization (GJO) [22], Subtraction-Average-Based Optimizer (SABO) [23], Sand Cat Swarm Optimization (SCSO) [24], Pelican Optimization Algorithm (POA) [25], Sine Cosine Algorithm (SCA) [26] and Osprey Optimization Algorithm (OOA) [9] are compared with the IOOA. Table 3 demonstrates each algorithm’s parameter settings.

### 4.2. Convergence Curve Comparison Analysis

**Figure 3:**
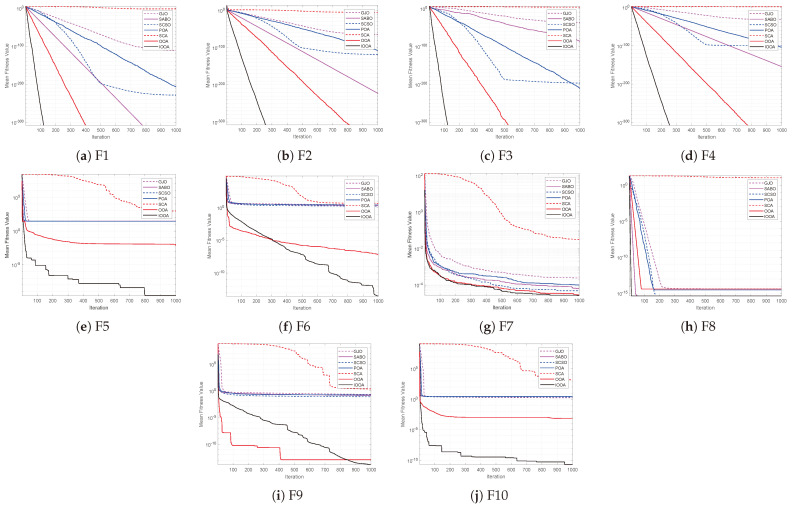
Benchmark test function convergence curves.

The convergence curves clearly show the convergence accuracy and speed of each algorithm and the algorithms’ performance in jumping out of the local extremes. In order to ensure the experimental fairness, the algorithms’ population size is set to 30, the maximum iterations is 1000, the dimension is 30, and each algorithm is run independently 30 times in order to avoid chance. Figure 3 gives the 10 convergence curves of the IOOA with the other six optimization algorithms corresponding to the test functions F1−F10. In the figure, the horizontal coordinate represents iterations, the vertical coordinate represents the functions’ average fitness value, and the convergence curve represents the average fitness value searched by the algorithm at the current iterations.

From Figure 3a–g, it can be seen that the IOOA has better optimization accuracy and convergence speed compared to the other six algorithms for single-peaked functions. When solving F1−F4, the IOOA improves the optimization accuracy by about 260 magnitude orders compared to the SCA, and by about 200 magnitude orders compared to the GJO. When solving F1 and F3, the IOOA’s optimization accuracy is improved by about 100 magnitude orders compared to the SCSO and POA, and the optimal value is found in about 120 iterations. When solving F2 and F4, the IOOA’s optimization accuracy is improved by about 200 orders of magnitude compared to SCSO and POA, and the optimal value can be found in about 250 iterations. For F1−F4, although the optimization accuracy of the IOOA is the same as the OOA, the convergence speed of the IOOA is faster than the OOA, which is a significant advantage. When solving F5 and F6, the IOOA has the ability to significantly leap out of the local optima, and the IOOA’s optimization accuracy is better compared to the other six algorithms.

From Figure 3h–j, for F8, the IOOA converges faster compared to the SABO and SCSO, and has higher optimization accuracy compared to the GJO, POA, SCA and OOA. For F9, the IOOA has significantly better optimization accuracy than the other six algorithms and is less likely to fall into local optima. For F10, the IOOA has a significant advantage in terms of higher search accuracy and faster convergence.

In summary, the IOOA has better convergence speed and optimization accuracy when solving the benchmark test functions. It can effectively prevent falling into local optima, and the optimization performance improves significantly, which illustrates the IOOA’s effectiveness and superiority.

### 4.3. Optimization Accuracy Comparison

In this section, the IOOA is selected to be tested against the other six comparison algorithms for optimization of the 10 benchmark functions in Table 1 when the dimensions are 30 or 50 or 100. Each algorithm’s population size is set to 30, the maximum number of iterations is 1000, and each algorithm is run independently 30 times. This paper selects the mean and standard deviation of the algorithm optimization as the evaluation metrics. The mean value reflects the algorithm optimization accuracy, and the standard deviation reflects the algorithm optimization stability. At the same time, this paper performs the Friedman test. Each algorithm’s results are ranked and algorithms with the same result are given an average ranking. In Table 4, Table 5 and Table 6, Rank-Count is the sum rankings, Ave-Rank is the average rankings, and Overall-Rank is the final ranking on the benchmark function.

Table 4 represents the test results for dimension 30, where the IOOA achieves the theoretically optimal solution when solving F1−F4, and the mean and standard deviation are both 0. Although the theoretical optimal solution is not achieved for F5−F10, the results are better than other algorithms, with the smallest mean and standard deviation, which indicates that the IOOA has good optimization accuracy and stability. Meanwhile, in Table 4, the Friedman test results show that the IOOA has the highest Ave-Rank and ranks first in Overall-Rank.

From Table 5 and Table 6, at dimension 50, the optimization accuracy of the IOOA for F6 and F9 is reduced compared to 30 dimensions, but it is still optimal compared with other algorithms. When solving other functions, it has almost no degradation in the optimization accuracy. At dimension 100,the IOOA’s optimization accuracy decreases for F5, F6 and F9, but it is still the best result, and on the rest of the functions, there is almost no change in the optimization accuracy compared to 30 dimensions. The Friedman test results in Table 5 and Table 6 show that the IOOA is still ranked first. As the dimension increases, the IOOA’s optimization performance does not degrade and the IOOA can solve some multidimensionalization problems.

In summary, compared with the other six algorithms, the IOOA has better optimization ability and stability when solving the benchmark test functions, and has obvious advantages.

### 4.4. Wilcoxon Rank-Sum Test

In order to comprehensively assess the IOOA’s reliability and superiority, this paper selects the Wilcoxon rank-sum test to further validate the significant difference in each algorithm’s experimental results. This paper selects the IOOA’s results running on the benchmark functions on three different dimensions. The IOOA performs the Wilcoxon rank-sum test with six other algorithms and calculates the test results. The significance level is 5%; when the *p*-value is less than 5%, it means that the difference between two algorithms is significant, and otherwise, it is not. The experiment results are shown in Table 7, where “NAN” shows that the algorithm has the same results as the IOOA, and “+”, “−” and “=” indicate that the IOOA’s performance is superior, inferior and equal to the comparison algorithms.

From Table 7, there is a significant difference between the IOOA and GJO, POA, SCA for F1–F10 at dimensions 30, 50 and 100. For F1–F4, the difference between the IOOA and OOA was not significant. For F1 and F8, the difference between the IOOA and SABO was not significant. For F8, the variability between the IOOA and SCSO is not significant. In general, the IOOA has significant advantages over other algorithms, further confirming statistically the IOOA’s validity and reliability.

### 4.5. The IOOA Solves CEC2017 Test Functions

In order to further verify the IOOA’s superiority and robustness and improve the test results’ reliability, the IOOA is tested with the GJO, SABO, SCSO, POA, SCA and OOA for optimization searching for the 15 CEC2017 test functions selected in Table 2. The experimental parameters set the population size to 30, the maximum iterations to 1000, the dimension to 30, and each algorithm is run independently 30 times. The specific optimization results are shown in Table 8.

The high complexity of the IEEE CEC2017 test functions makes it difficult to search for the objective functions’ optimal value, which can only be compared with other algorithms to find the relative optimal value. From Table 8, the IOOA achieves the relative optimal average for F11, F13–F25, and for F12, although it is inferior to the POA, it achieves the optimal results except the POA, and has the best overall optimization-seeking effect, which is more advantageous compared with other algorithms, showing that the IOOA has a better optimization-seeking ability. This further verifies the IOOA’s superiority. The IOOA achieved the minimum standard deviation for F11–F13 and F15–F25, which is slightly smaller than the SCA for F14, and two algorithms have a small difference in numerical results. Overall, the IOOA achieved the optimal standard deviation for most of the functions with good overall stability, which further indicates that the IOOA has good robustness. Meanwhile, the Friedman test results in Table 8 shows that the IOOA has the highest Ave-Rank and ranks first in Overall-Rank.

## 5. Engineering Design Problem

In order to further validate the IOOA’s feasibility and effectiveness, this paper selects the engineering optimization problem of three-bar truss design and compares the IOOA with the other six optimization algorithms in Section 5 for validation.

**Figure 4:**
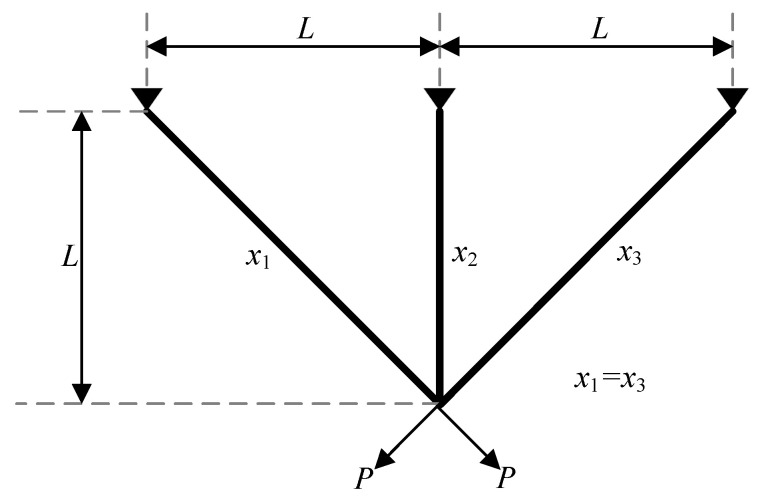
A schematic model of three-bar truss.

The purpose of the three-bar truss design problem is to minimize the volume of the three-rod truss by adjusting the cross-sectional area ($x_{1},x_{2}$) and to make the bearing capacity of each truss (σ) satisfy the constraint conditions. Its structural design is shown in Figure 4. Equation (17) shows a mathematical model of the problem.

(17) $$\begin{array}{c} \min f\left(x\right)=\left(2\sqrt{2}x_{1}+x_{2}\right)\cdot L\\ g_{1}\left(x\right)=\frac{\sqrt{2}x_{1}+x_{2}}{\sqrt{2}x_{1}^{2}+2x_{1}x_{2}}P-\sigma \leq 0\\ g_{2}\left(x\right)=\frac{x_{2}}{\sqrt{2}x_{1}^{2}+2x_{1}x_{2}}P-\sigma \leq 0\\ g_{3}\left(x\right)=\frac{1}{\sqrt{2}x_{2}+x_{1}}P-\sigma \leq 0\\ 0\leq x_{1},x_{2}\leq 1\\ L=100\;\mathrm{cm},P=2\;{\mathrm{kN}/\mathrm{cm}}^{2},\sigma =2\;{\mathrm{kN}/\mathrm{cm}}^{2} \end{array}$$

Table 9 shows the results of solving the three-bar truss design problem. From Table 9, the IOOA obtains the optimal value of 263.895849, and the volume of the three-bar truss is minimized when the cross-sectional areas are 0.788764 and 0.407998, respectively. It indicates that the IOOA has better optimization-seeking performance in solving the three-bar truss design problem and has some advantages.

## 6. Conclusions

Based on the OOA, this paper proposes an improved osprey optimization algorithm (IOOA) with multi-strategy fusion.The IOOA introduces Fuch chaotic mapping to increase the population diversity at the early stage of algorithm iteration; introduces an adaptive weighting factor to improve the convergence speed and accuracy at the exploration stage of the algorithm; incorporates Cauchy’s variational operator to prevent osprey individuals from falling into the local optima at the algorithm’s exploitation stage; and finally incorporates the Warner mechanism of the sparrow search algorithm to coordinate the algorithm’s local exploration and global search capability. The IOOA is verified to have better optimization performance and stronger robustness by 10 benchmark test functions, Friedman ranking test and Wilcoxon rank-sum test. Then, the IOOA’s superiority is further verified by solving 15 CEC2017 test functions. Finally, the IOOA is applied to the three-bar truss design problem to verify its applicability and reliability in solving practical engineering problems. In the future, the IOOA can be further applied to other fields. 

## Figures and Tables

**Table 1:** Benchmark functions.

Function	Range	Min
F1(Sphere)	[−100, 100]	0
F2(Schwefel 2.22)	[−10, 10]	0
F3(Schwefel 1.2)	[−100, 100]	0
F4(Schwefel 2.21)	[−100, 100]	0
F5(Rosenbrock)	[−30, 30]	0
F6(Step)	[−100, 100]	0
F7(Quartic)	[−1.28, 1.28]	0
F8(Ackley)	[−32, 32]	0
F9(Penalized 1.1)	[−50, 50]	0
F10(Penalized 1.2)	[−50, 50]	0

**Table 2:** CEC2017 test functions.

Function	Range	Min
F11(CEC-1)	[−100, 100]	100
F12(CEC-3)	[−100, 100]	300
F13(CEC-4)	[−100, 100]	400
F14(CEC-8)	[−100, 100]	800
F15(CEC-11)	[−100, 100]	1100
F16(CEC-12)	[−100, 100]	1200
F17(CEC-13)	[−100, 100]	1300
F18(CEC-15)	[−100, 100]	1500
F19(CEC-19)	[−100, 100]	1900
F20(CEC-22)	[−100, 100]	2200
F21(CEC-25)	[−100, 100]	2500
F22(CEC-26)	[−100, 100]	2600
F23(CEC-28)	[−100, 100]	2800
F24(CEC-29)	[−100, 100]	2900
F25(CEC-30)	[−100, 100]	3000

**Table 3:** Parameter settings.

Algorithm	Parameters
IOOA	$r\in \left[\begin{array}{c} 0,1 \end{array}\right]$, *I* = 1 or 2, $w\in \left[\begin{array}{c} 0,1 \end{array}\right]$
GJO	$c_{1}=1.5$, $r\in \left[\begin{array}{c} 0,1 \end{array}\right]$, μ∈[0,1], $v\in \left[\begin{array}{c} 0,1 \end{array}\right]$, β=1.5
SABO	*v* = 1 or 2, $r\in \left[\begin{array}{c} 0,1 \end{array}\right]$
SCSO	$s_{M}=2$
POA	*I* = 1 or 2, *R* = 0.2
SCA	*a* = 2, $r_{2}\in \left[0,2\pi \right]$, $r_{3}\in \left[\begin{array}{c} 0,2 \end{array}\right]$, $r_{4}\in \left[\begin{array}{c} 0,1 \end{array}\right]$
OOA	$r\in \left[\begin{array}{c} 0,1 \end{array}\right]$, *I* = 1 or 2

**Table 4:** Benchmark function optimization results (D = 30).

Function	Index	GJO	SABO	SCSO	POA	SCA	OOA	IOOA
F1	Mean	$2.51\times 10^{-111}$	0	$4.52\times 10^{-229}$	$2.98\times 10^{-207}$	$1.81\times 10^{-2}$	0	**0**
	Std	$1.21\times 10^{-110}$	0	0	0	$6.91\times 10^{-2}$	0	**0**
	Rank	6	2	4	5	7	2	2
F2	Mean	$1.52\times 10^{-65}$	$6.44\times 10^{-224}$	$7.29\times 10^{-119}$	$2.52\times 10^{-108}$	$9.54\times 10^{-6}$	0	**0**
	Std	$3.87\times 10^{-65}$	0	$3.97\times 10^{-118}$	$1.11\times 10^{-107}$	$1.42\times 10^{-5}$	0	**0**
	Rank	6	3	4	5	7	1.5	1.5
F3	Mean	$1.45\times 10^{-37}$	$3.62\times 10^{-87}$	$2.21\times 10^{-197}$	$1.21\times 10^{-211}$	$3.77\times 10^{3}$	0	**0**
	Std	$5.98\times 10^{-37}$	$1.80\times 10^{-86}$	0	0	$2.63\times 10^{3}$	0	**0**
	Rank	6	5	4	3	7	1.5	1.5
F4	Mean	$4.21\times 10^{-33}$	$1.61\times 10^{-155}$	$2.28\times 10^{-101}$	$5.38\times 10^{-105}$	$2.38\times 10^{1}$	0	**0**
	Std	$1.11\times 10^{-32}$	$2.93\times 10^{-155}$	$6.06\times 10^{-101}$	$2.91\times 10^{-104}$	$1.12\times 10^{1}$	0	**0**
	Rank	6	3	5	4	7	1.5	1.5
F5	Mean	$2.78\times 10^{1}$	$2.82\times 10^{1}$	$2.80\times 10^{1}$	$2.76\times 10^{1}$	$9.38\times 10^{-2}$	$7.18\times 10^{-3}$	$2.53\times 10^{-10}$
	Std	$7.46\times 10^{-1}$	$5.54\times 10^{-1}$	$6.99\times 10^{-1}$	$9.49\times 10^{-1}$	$3.52\times 10^{3}$	$2.37\times 10^{-2}$	$7.74\times 10^{-10}$
	Rank	4	6	5	3	7	2	1
F6	Mean	2.62	1.97	1.64	2.59	4.55	$7.00\times 10^{-8}$	$2.89\times 10^{-14}$
	Std	$4.81\times 10^{-1}$	$5.22\times 10^{-1}$	$5.38\times 10^{-1}$	$5.41\times 10^{-1}$	$4.92\times 10^{-1}$	$2.24\times 10^{-7}$	$7.38\times 10^{-14}$
	Rank	5	4	3	6	7	2	1
F7	Mean	$2.37\times 10^{-4}$	$6.33\times 10^{-5}$	$4.80\times 10^{-5}$	$1.01\times 10^{-4}$	$3.11\times 10^{-2}$	$2.80\times 10^{-5}$	$2.65\times 10^{-5}$
	Std	$1.37\times 10^{-4}$	$4.88\times 10^{-5}$	$5.28\times 10^{-5}$	$6.72\times 10^{-5}$	$3.15\times 10^{-2}$	$2.31\times 10^{-5}$	$2.12\times 10^{-5}$
	Rank	6	4	3	5	7	2	1
F8	Mean	$4.23\times 10^{-15}$	$4.44\times 10^{-16}$	$4.44\times 10^{-16}$	$3.05\times 10^{3}$	$1.08\times 10^{1}$	$4.00\times 10^{-15}$	$4.44\times 10^{-16}$
	Std	$9.01\times 10^{-16}$	0	0	$1.60\times 10^{-15}$	9.57	0	**0**
	Rank	6	2	2	4	7	5	2
F9	Mean	$2.18\times 10^{-1}$	$1.31\times 10^{-1}$	$7.53\times 10^{-2}$	$1.70\times 10^{-1}$	2.31	$1.26\times 10^{-13}$	$1.49\times 10^{-14}$
	Std	$1.19\times 10^{-1}$	$5.20\times 10^{-2}$	$4.13\times 10^{-2}$	$5.51\times 10^{-2}$	2.65	$3.36\times 10^{-13}$	$7.61\times 10^{-14}$
	Rank	6	4	3	5	7	1	2
F10	Mean	1.66	2.76	2.31	2.74	$1.50\times 10^{3}$	$7.33\times 10^{-4}$	$2.00\times 10^{-11}$
	Std	$2.41\times 10^{-1}$	$4.83\times 10^{-1}$	$3.87\times 10^{-1}$	$3.57\times 10^{-1}$	$7.70\times 10^{3}$	$2.79\times 10^{-3}$	$8.89\times 10^{-11}$
	Rank	3	6	4	5	7	2	1
Rank-Count		54	39	37	45	70	20.5	14.5
Ave-Rank		5.4	3.9	3.7	4.5	7.0	2.05	1.45
Overall-Rank		6	4	3	5	7	2	1

**Table 5:** Benchmark function optimization results (D = 50).

Function	Index	GJO	SABO	SCSO	POA	SCA	OOA	IOOA
F1	Mean	$7.35\times 10^{-84}$	0	$4.64\times 10^{-221}$	$3.80\times 10^{-208}$	$1.27\times 10^{2}$	0	**0**
	Std	$2.31\times 10^{-83}$	0	0	0	$3.27\times 10^{2}$	0	**0**
	Rank	6	2	4	5	7	2	2
F2	Mean	$8.76\times 10^{-51}$	$5.04\times 10^{-227}$	$8.24\times 10^{-118}$	$7.32\times 10^{-104}$	$1.83\times 10^{-2}$	0	**0**
	Std	$2.96\times 10^{-50}$	0	$2.24\times 10^{-117}$	$4.01\times 10^{-103}$	$6.62\times 10^{-2}$	0	**0**
	Rank	6	3	4	5	7	1.5	1.5
F3	Mean	$2.27\times 10^{-22}$	$3.69\times 10^{-42}$	$8.35\times 10^{-188}$	$1.23\times 10^{-207}$	$3.75\times 10^{4}$	0	**0**
	Std	$1.15\times 10^{-21}$	$2.02\times 10^{-41}$	0	0	$1.26\times 10^{4}$	0	**0**
	Rank	6	5	4	3	7	1.5	1.5
F4	Mean	$9.01\times 10^{-16}$	$2.47\times 10^{-152}$	$3.88\times 10^{-99}$	$5.41\times 10^{-104}$	$6.14\times 10^{1}$	0	**0**
	Std	$4.93\times 10^{-15}$	$5.36\times 10^{-152}$	$2.02\times 10^{-98}$	$2.83\times 10^{-103}$	7.05	0	**0**
	Rank	6	3	5	4	7	1.5	1.5
F5	Mean	$4.77\times 10^{1}$	$4.83\times 10^{1}$	$4.83\times 10^{1}$	$4.81\times 10^{1}$	$1.39\times 10^{6}$	$7.49\times 10^{-3}$	$3.65\times 10^{-10}$
	Std	$8.02\times 10^{-1}$	$4.54\times 10^{-1}$	$5.94\times 10^{-1}$	$6.91\times 10^{-1}$	$1.99\times 10^{6}$	$1.94\times 10^{-2}$	$1.95\times 10^{-9}$
	Rank	3	5	6	4	7	2	1
F6	Mean	6.02	5.32	4.80	5.32	$1.22\times 10^{2}$	$3.80\times 10^{-7}$	$9.21\times 10^{-9}$
	Std	$6.71\times 10^{-1}$	$6.25\times 10^{-1}$	$9.31\times 10^{-1}$	$8.00\times 10^{-1}$	$3.48\times 10^{2}$	$6.76\times 10^{-7}$	$2.39\times 10^{-8}$
	Rank	6	4	3	5	7	2	1
F7	Mean	$4.15\times 10^{-4}$	$7.75\times 10^{-5}$	$1.07\times 10^{-4}$	$1.01\times 10^{-4}$	$5.40\times 10^{-1}$	$3.45\times 10^{-5}$	$2.30\times 10^{-5}$
	Std	$5.57\times 10^{-4}$	$5.95\times 10^{-5}$	$1.04\times 10^{-4}$	$6.65\times 10^{-5}$	$7.46\times 10^{-1}$	$2.91\times 10^{-5}$	$1.98\times 10^{-5}$
	Rank	6	3	4	5	7	2	1
F8	Mean	$6.84\times 10^{-15}$	$4.44\times 10^{-16}$	$4.44\times 10^{-16}$	$3.52\times 10^{-15}$	$1.66\times 10^{1}$	$4.00\times 10^{-15}$	$4.44\times 10^{-16}$
	Std	$1.45\times 10^{-15}$	0	0	$1.23\times 10^{-15}$	7.26	0	**0**
	Rank	6	2	2	4	7	5	2
F9	Mean	$3.97\times 10^{-1}$	$2.74\times 10^{-1}$	$1.74\times 10^{-1}$	$2.46\times 10^{-1}$	$2.90\times 10^{6}$	$2.21\times 10^{-8}$	$6.38\times 10^{-12}$
	Std	$8.69\times 10^{-2}$	$6.44\times 10^{-2}$	$7.45\times 10^{-2}$	$5.77\times 10^{-2}$	$5.50\times 10^{6}$	$5.08\times 10^{-8}$	$1.74\times 10^{-11}$
	Rank	6	5	3	4	7	2	1
F10	Mean	3.49	4.97	4.62	4.91	$3.76\times 10^{6}$	$4.01\times 10^{-3}$	$1.54\times 10^{-11}$
	Std	$2.27\times 10^{-1}$	$2.94\times 10^{-2}$	$1.64\times 10^{-1}$	$2.28\times 10^{-1}$	$6.55\times 10^{6}$	$1.61\times 10^{-2}$	$6.77\times 10^{-11}$
	Rank	3	6	4	5	7	2	1
Rank-Count		54	38	39	44	70	21.5	13.5
Ave-Rank		5.4	3.8	3.9	4.4	70	2.15	1.35
Overall-Rank		6	3	4	5	7	2	1

**Table 6:** Benchmark function optimization results (D = 100).

Function	Index	GJO	SABO	SCSO	POA	SCA	OOA	IOOA
F1	Mean	$3.27\times 10^{-60}$	0	$1.66\times 10^{-213}$	$1.33\times 10^{-208}$	$5.42\times 10^{3}$	0	**0**
	Std	$5.18\times 10^{-60}$	0	0	0	$3.72\times 10^{3}$	0	**0**
	Rank	6	2	4	5	7	2	2
F2	Mean	$4.55\times 10^{-37}$	$1.83\times 10^{-231}$	$4.06\times 10^{-106}$	$2.53\times 10^{-111}$	2.43	0	**0**
	Std	$4.25\times 10^{-37}$	0	$1.91\times 10^{-105}$	$1.16\times 10^{-110}$	3.11	0	**0**
	Rank	6	3	5	4	7	1.5	1.5
F3	Mean	$2.63\times 10^{-4}$	$1.17\times 10^{-17}$	$2.39\times 10^{-183}$	$1.00\times 10^{-204}$	$2.12\times 10^{5}$	0	**0**
	Std	$1.42\times 10^{-3}$	$6.33\times 10^{-17}$	0	0	$4.35\times 10^{4}$	0	**0**
	Rank	6	5	4	3	7	1.5	1.5
F4	Mean	1.22	$7.42\times 10^{-148}$	$1.76\times 10^{-97}$	$1.94\times 10^{-105}$	$8.55\times 10^{1}$	0	**0**
	Std	4.26	$3.67\times 10^{-147}$	$6.42\times 10^{-97}$	$6.44\times 10^{-105}$	2.97	0	**0**
	Rank	6	3	5	4	7	1.5	1.5
F5	Mean	$9.80\times 10^{1}$	$9.85\times 10^{1}$	$9.85\times 10^{1}$	$9.83\times 10^{1}$	$6.34\times 10^{7}$	$2.42\times 10^{-1}$	$8.46\times 10^{-9}$
	Std	$7.40\times 10^{-1}$	$2.34\times 10^{-1}$	$3.48\times 10^{-1}$	$5.12\times 10^{-1}$	$4.07\times 10^{7}$	$9.00\times 10^{-1}$	$2.39\times 10^{-8}$
	Rank	4	5	6	3	7	2	1
F6	Mean	$1.71\times 10^{1}$	$1.47\times 10^{1}$	$1.31\times 10^{1}$	$1.37\times 10^{1}$	$5.20\times 10^{3}$	$1.10\times 10^{-4}$	$6.64\times 10^{-8}$
	Std	$8.06\times 10^{-1}$	$9.81\times 10^{-1}$	1.47	1.15	$3.6\times 10^{3}$	$2.67\times 10^{-4}$	$1.87\times 10^{-7}$
	Rank	6	5	3	4	7	2	1
F7	Mean	$4.63\times 10^{-4}$	$7.21\times 10^{-5}$	$9.12\times 10^{-5}$	$9.19\times 10^{-5}$	$6.50\times 10^{1}$	$3.68\times 10^{-5}$	$2.51\times 10^{-5}$
	Std	$2.99\times 10^{-4}$	$5.51\times 10^{-5}$	$9.41\times 10^{-5}$	$5.67\times 10^{-5}$	$4.38\times 10^{1}$	$2.81\times 10^{-5}$	$2.66\times 10^{-5}$
	Rank	6	3	4	5	7	2	1
F8	Mean	$9.21\times 10^{-15}$	$4.44\times 10^{-16}$	$4.44\times 10^{-16}$	$3.17\times 10^{-15}$	$1.97\times 10^{1}$	$4.00\times 10^{-15}$	$4.44\times 10^{-16}$
	Std	$2.91\times 10^{-15}$	0	0	$1.53\times 10^{-15}$	3.28	0	**0**
	Rank	6	2	2	4	7	5	2
F9	Mean	$5.96\times 10^{-1}$	$4.43\times 10^{-1}$	$2.96\times 10^{-1}$	$3.86\times 10^{-1}$	$1.49\times 10^{8}$	$1.15\times 10^{-6}$	$2.43\times 10^{-12}$
	Std	$5.14\times 10^{-2}$	$8.76\times 10^{-2}$	$5.84\times 10^{-2}$	$5.69\times 10^{-2}$	$1.02\times 10^{8}$	$1.42\times 10^{-6}$	$1.03\times 10^{-11}$
	Rank	6	5	3	4	7	2	1
F10	Mean	8.54	9.95	9.66	9.95	$2.82\times 10^{8}$	$1.15\times 10^{-3}$	$1.31\times 10^{-11}$
	Std	$2.78\times 10^{-1}$	$7.07\times 10^{-3}$	$9.90\times 10^{-2}$	$6.18\times 10^{-3}$	$1.98\times 10^{8}$	$3.38\times 10^{-3}$	$4.05\times 10^{-11}$
	Rank	3	6	4	5	7	2	1
Rank-Count		55	39	40	41	70	21.5	13.5
Ave-Rank		5.5	3.9	4.0	4.1	7.0	2.15	1.35
Overall-Rank		6	3	4	5	7	2	1

**Table 7:** Wilcoxon rank-sum test results.

dim	Function	GJO	SABO	SCSO	POA	SCA	OOA
D = 30	F1	$1.21\times 10^{-12}$	NAN	$1.21\times 10^{-12}$	$1.21\times 10^{-12}$	$1.21\times 10^{-12}$	NAN
	F2	$1.21\times 10^{-12}$	$1.21\times 10^{-12}$	$1.21\times 10^{-12}$	$1.21\times 10^{-12}$	$1.21\times 10^{-12}$	NAN
	F3	$1.21\times 10^{-12}$	$1.21\times 10^{-12}$	$1.21\times 10^{-12}$	$1.21\times 10^{-12}$	$1.21\times 10^{-12}$	NAN
	F4	$1.21\times 10^{-12}$	$1.21\times 10^{-12}$	$1.21\times 10^{-12}$	$1.21\times 10^{-12}$	$1.21\times 10^{-12}$	NAN
	F5	$3.02\times 10^{-11}$	$3.02\times 10^{-11}$	$3.02\times 10^{-11}$	$3.02\times 10^{-11}$	$3.02\times 10^{-11}$	$1.96\times 10^{-11}$
	F6	$3.02\times 10^{-11}$	$3.02\times 10^{-11}$	$3.02\times 10^{-11}$	$3.02\times 10^{-11}$	$3.02\times 10^{-11}$	$3.25\times 10^{-6}$
	F7	$2.61\times 10^{-10}$	$3.37\times 10^{-4}$	$1.91\times 10^{-3}$	$1.86\times 10^{-6}$	$3.02\times 10^{-11}$	$7.62\times 10^{-3}$
	F8	$4.16\times 10^{-14}$	NAN	NAN	$5.36\times 10^{-9}$	$1.21\times 10^{-12}$	$1.69\times 10^{-14}$
	F9	$3.02\times 10^{-11}$	$3.02\times 10^{-11}$	$3.02\times 10^{-11}$	$3.02\times 10^{-11}$	$3.02\times 10^{-11}$	$3.21\times 10^{-3}$
	F10	$3.02\times 10^{-11}$	$3.02\times 10^{-11}$	$3.02\times 10^{-11}$	$3.02\times 10^{-11}$	$3.02\times 10^{-11}$	$7.41\times 10^{-4}$
	+/=/−	10/0/0	8/2/0	9/1/0	10/0/0	10/0/0	6/4/0
D = 50	F1	$1.21\times 10^{-12}$	NAN	$1.21\times 10^{-12}$	$1.21\times 10^{-12}$	$1.21\times 10^{-12}$	NAN
	F2	$1.21\times 10^{-12}$	$1.21\times 10^{-12}$	$1.21\times 10^{-12}$	$1.21\times 10^{-12}$	$1.21\times 10^{-12}$	NAN
	F3	$1.21\times 10^{-12}$	$1.21\times 10^{-12}$	$1.21\times 10^{-12}$	$1.21\times 10^{-12}$	$1.21\times 10^{-12}$	NAN
	F4	$1.21\times 10^{-12}$	$1.21\times 10^{-12}$	$1.21\times 10^{-12}$	$1.21\times 10^{-12}$	$1.21\times 10^{-12}$	NAN
	F5	$3.02\times 10^{-11}$	$3.02\times 10^{-11}$	$3.02\times 10^{-11}$	$3.02\times 10^{-11}$	$3.02\times 10^{-11}$	$2.80\times 10^{-11}$
	F6	$3.02\times 10^{-11}$	$3.02\times 10^{-11}$	$3.02\times 10^{-11}$	$3.02\times 10^{-11}$	$3.02\times 10^{-11}$	$2.56\times 10^{-8}$
	F7	$4.50\times 10^{-11}$	$4.74\times 10^{-6}$	$7.22\times 10^{-6}$	$5.09\times 10^{-8}$	$3.02\times 10^{-11}$	$8.24\times 10^{-2}$
	F8	$1.55\times 10^{-13}$	NAN	NAN	$1.97\times 10^{-11}$	$1.21\times 10^{-12}$	$1.69\times 10^{-14}$
	F9	$3.02\times 10^{-11}$	$3.02\times 10^{-11}$	$3.02\times 10^{-11}$	$3.02\times 10^{-11}$	$3.02\times 10^{-11}$	$7.38\times 10^{-11}$
	F10	$3.02\times 10^{-11}$	$3.02\times 10^{-11}$	$3.02\times 10^{-11}$	$3.02\times 10^{-11}$	$3.02\times 10^{-11}$	$5.38\times 10^{-11}$
	+/=/−	10/0/0	8/2/0	9/1/0	10/0/0	10/0/0	6/4/0
D = 100	F1	$1.21\times 10^{-12}$	NAN	$1.21\times 10^{-12}$	$1.21\times 10^{-12}$	$1.21\times 10^{-12}$	NAN
	F2	$1.21\times 10^{-12}$	$1.21\times 10^{-12}$	$1.21\times 10^{-12}$	$1.21\times 10^{-12}$	$1.21\times 10^{-12}$	NAN
	F3	$1.21\times 10^{-12}$	$1.21\times 10^{-12}$	$1.21\times 10^{-12}$	$1.21\times 10^{-12}$	$1.21\times 10^{-12}$	NAN
	F4	$1.21\times 10^{-12}$	$1.21\times 10^{-12}$	$1.21\times 10^{-12}$	$1.21\times 10^{-12}$	$1.21\times 10^{-12}$	NAN
	F5	$3.02\times 10^{-11}$	$3.02\times 10^{-11}$	$3.02\times 10^{-11}$	$3.02\times 10^{-11}$	$3.02\times 10^{-11}$	$2.80\times 10^{-11}$
	F6	$3.02\times 10^{-11}$	$3.02\times 10^{-11}$	$3.02\times 10^{-11}$	$3.02\times 10^{-11}$	$3.02\times 10^{-11}$	$3.22\times 10^{-11}$
	F7	$4.50\times 10^{-11}$	$4.86\times 10^{-3}$	$3.51\times 10^{-2}$	$2.28\times 10^{-5}$	$3.02\times 10^{-11}$	$3.92\times 10^{-2}$
	F8	$2.54\times 10^{-13}$	NAN	NAN	$1.47\times 10^{-9}$	$1.21\times 10^{-12}$	$1.69\times 10^{-14}$
	F9	$3.02\times 10^{-11}$	$3.02\times 10^{-11}$	$3.02\times 10^{-11}$	$3.02\times 10^{-11}$	$3.02\times 10^{-11}$	$2.92\times 10^{-11}$
	F10	$3.02\times 10^{-11}$	$3.02\times 10^{-11}$	$3.02\times 10^{-11}$	$3.02\times 10^{-11}$	$3.02\times 10^{-11}$	$2.40\times 10^{-11}$
	+/=/−	10/0/0	8/2/0	9/1/0	10/0/0	10/0/0	6/4/0

**Table 8:** CEC2017 test function optimization results.

Function	Index	GJO	SABO	SCSO	POA	SCA	OOA	IOOA
F11	Mean	$1.23\times 10^{10}$	$9.84\times 10^{9}$	$8.33\times 10^{9}$	$1.48\times 10^{10}$	$1.88\times 10^{10}$	$5.77\times 10^{10}$	$4.51\times 10^{3}$
	Std	$3.71\times 10^{9}$	$3.41\times 10^{9}$	$3.65\times 10^{9}$	$5.65\times 10^{9}$	$2.81\times 10^{9}$	$8.54\times 10^{9}$	$4.97\times 10^{3}$
	Rank	4	3	2	5	6	7	1
F12	Mean	$5.59\times 10^{4}$	$5.45\times 10^{4}$	$5.05\times 10^{4}$	$3.28\times 10^{4}$	$7.30\times 10^{4}$	$9.01\times 10^{4}$	$3.58\times 10^{4}$
	Std	$1.05\times 10^{4}$	$1.02\times 10^{4}$	$1.10\times 10^{4}$	$8.77\times 10^{3}$	$1.40\times 10^{4}$	$7.84\times 10^{3}$	$7.08\times 10^{3}$
	Rank	5	4	3	1	6	7	2
F13	Mean	$1.26\times 10^{3}$	$1.75\times 10^{3}$	$9.93\times 10^{2}$	$1.93\times 10^{3}$	$2.45\times 10^{3}$	$1.52\times 10^{4}$	$4.93\times 10^{2}$
	Std	$5.85\times 10^{2}$	$1.27\times 10^{3}$	$4.91\times 10^{2}$	$1.20\times 10^{3}$	$7.26\times 10^{2}$	$3.70\times 10^{3}$	$2.39\times 10^{1}$
	Rank	3	4	2	5	6	7	1
F14	Mean	$9.75\times 10^{2}$	$1.08\times 10^{3}$	$9.97\times 10^{2}$	$9.87\times 10^{2}$	$1.09\times 10^{3}$	$1.14\times 10^{3}$	$9.66\times 10^{2}$
	Std	$4.39\times 10^{1}$	$3.33\times 10^{1}$	$2.87\times 10^{1}$	$2.24\times 10^{1}$	$2.08\times 10^{1}$	$2.55\times 10^{1}$	$2.09\times 10^{1}$
	Rank	2	5	4	3	6	7	1
F15	Mean	$3.05\times 10^{3}$	$4.88\times 10^{3}$	$2.72\times 10^{3}$	$2.07\times 10^{3}$	$3.12\times 10^{3}$	$9.08\times 10^{3}$	$1.27\times 10^{3}$
	Std	$1.46\times 10^{3}$	$1.47\times 10^{3}$	$1.57\times 10^{3}$	$6.98\times 10^{3}$	$7.10\times 10^{2}$	$2.20\times 10^{3}$	$7.51\times 10^{1}$
	Rank	4	6	3	2	5	7	1
F16	Mean	$1.09\times 10^{9}$	$5.87\times 10^{8}$	$1.92\times 10^{8}$	$1.34\times 10^{9}$	$2.13\times 10^{9}$	$1.26\times 10^{10}$	$2.81\times 10^{6}$
	Std	$9.31\times 10^{8}$	$4.48\times 10^{8}$	$3.29\times 10^{8}$	$1.28\times 10^{9}$	$6.06\times 10^{8}$	$3.60\times 10^{9}$	$4.53\times 10^{6}$
	Rank	4	3	2	5	6	7	1
F17	Mean	$3.31\times 10^{8}$	$6.14\times 10^{7}$	$7.62\times 10^{7}$	$1.32\times 10^{7}$	$8.33\times 10^{8}$	$9.69\times 10^{9}$	$2.68\times 10^{4}$
	Std	$7.49\times 10^{8}$	$1.31\times 10^{8}$	$1.50\times 10^{8}$	$3.39\times 10^{7}$	$4.26\times 10^{8}$	$4.45\times 10^{9}$	$1.85\times 10^{4}$
	Rank	5	4	3	2	6	7	1
F18	Mean	$6.79\times 10^{6}$	$6.44\times 10^{5}$	$9.91\times 10^{5}$	$4.11\times 10^{4}$	$4.56\times 10^{7}$	$6.40\times 10^{8}$	$5.36\times 10^{3}$
	Std	$1.36\times 10^{7}$	$9.01\times 10^{5}$	$3.63\times 10^{6}$	$2.42\times 10^{4}$	$2.62\times 10^{7}$	$6.56\times 10^{8}$	$4.69\times 10^{3}$
	Rank	5	4	3	2	6	7	1
F19	Mean	$2.43\times 10^{7}$	$5.57\times 10^{6}$	$7.41\times 10^{6}$	$1.35\times 10^{6}$	$7.57\times 10^{7}$	$7.63\times 10^{8}$	$8.49\times 10^{3}$
	Std	$4.30\times 10^{7}$	$6.98\times 10^{6}$	$2.66\times 10^{7}$	$1.79\times 10^{6}$	$3.28\times 10^{7}$	$5.72\times 10^{8}$	$6.78\times 10^{3}$
	Rank	5	4	3	2	6	7	1
F20	Mean	$5.62\times 10^{3}$	$3.82\times 10^{3}$	$4.46\times 10^{3}$	$4.89\times 10^{3}$	$9.45\times 10^{3}$	$9.43\times 10^{3}$	$2.81\times 10^{3}$
	Std	$2.39\times 10^{3}$	$1.55\times 10^{3}$	$1.79\times 10^{3}$	$1.65\times 10^{3}$	$1.70\times 10^{3}$	$9.31\times 10^{2}$	$6.74\times 10^{2}$
	Rank	5	2	3	4	7	6	1
F21	Mean	$3.23\times 10^{3}$	$3.27\times 10^{3}$	$3.13\times 10^{3}$	$3.30\times 10^{3}$	$3.46\times 10^{3}$	$5.02\times 10^{3}$	$2.90\times 10^{3}$
	Std	$1.52\times 10^{2}$	$1.37\times 10^{2}$	$1.04\times 10^{2}$	$1.86\times 10^{2}$	$1.78\times 10^{2}$	$4.53\times 10^{2}$	$1.64\times 10^{1}$
	Rank	3	5	2	4	6	7	1
F22	Mean	$6.01\times 10^{3}$	$8.05\times 10^{3}$	$6.66\times 10^{3}$	$7.03\times 10^{3}$	$7.49\times 10^{3}$	$1.17\times 10^{4}$	$4.18\times 10^{3}$
	Std	$6.70\times 10^{2}$	$8.29\times 10^{2}$	$1.00\times 10^{3}$	$1.52\times 10^{3}$	$1.92\times 10^{3}$	$1.05\times 10^{3}$	$3.33\times 10^{2}$
	Rank	2	6	3	4	5	7	1
F23	Mean	$3.93\times 10^{3}$	$4.03\times 10^{3}$	$3.62\times 10^{3}$	$4.05\times 10^{3}$	$4.17\times 10^{3}$	$7.48\times 10^{3}$	$3.22\times 10^{3}$
	Std	$3.30\times 10^{2}$	$3.72\times 10^{2}$	$1.75\times 10^{2}$	$4.00\times 10^{2}$	$2.41\times 10^{2}$	$8.04\times 10^{2}$	$1.93\times 10^{1}$
	Rank	3	5	2	4	6	7	1
F24	Mean	$4.26\times 10^{3}$	$5.69\times 10^{3}$	$4.51\times 10^{3}$	$4.51\times 10^{3}$	$5.13\times 10^{3}$	$9.05\times 10^{3}$	$4.03\times 10^{3}$
	Std	$2.61\times 10^{2}$	$5.74\times 10^{2}$	$3.60\times 10^{2}$	$3.17\times 10^{2}$	$2.53\times 10^{2}$	$3.59\times 10^{3}$	$2.45\times 10^{2}$
	Rank	2	6	4	3	5	7	1
F25	Mean	$3.59\times 10^{7}$	$2.71\times 10^{7}$	$1.60\times 10^{7}$	$1.21\times 10^{7}$	$1.55\times 10^{8}$	$1.70\times 10^{9}$	$3.52\times 10^{5}$
	Std	$2.84\times 10^{7}$	$2.36\times 10^{7}$	$1.45\times 10^{7}$	$7.33\times 10^{6}$	$7.10\times 10^{7}$	$1.26\times 10^{9}$	$8.52\times 10^{5}$
	Rank	5	4	3	2	6	7	1
Rank-Count		57	65	42	48	88	104	16
Ave-Rank		3.80	4.33	2.80	3.20	5.87	6.93	1.07
Overall-Rank		4	5	2	3	6	7	1

**Table 9:** Experimental results of different algorithms for solving the three-bar truss design problem.

Algorithm	Parameters		Best
	$x_{1}$	$x_{2}$	
GJO	0.792652	0.397171	263.913033
SABO	0.782390	0.427893	264.082722
SCSO	0.784455	0.420320	263.909287
POA	0.411249	0.411249	263.896682
SCA	0.796310	0.391495	264.379949
OOA	0.747933	0.537742	265.321646
IOOA	0.788764	0.407998	263.895849

## Data Availability

The data are contained within the article.
